# The Prevalence Rate of *Helicobacter pylori* Infection in, Chronic Otitis Media With Effusion Patients

**DOI:** 10.5812/jjm.15694

**Published:** 2014-03-01

**Authors:** Nader Saki, Ali Reza Samarbaf Zadeh, Reza Sheikhpour Jonaky, Seyed Mahdi Noori, Gholam Abbas Kayedani, Soheila Nikakhlagh

**Affiliations:** 1 Health Research Institute, Infectious and Tropical Diseases Research Center, Ahvaz Jundishapur University of Medical Sciences, Ahvaz, IR Iran; 2 Hearing and Speech Research Centre, Ahvaz Jundishapur University of Medical Sciences, Ahvaz, IR Iran

**Keywords:** Otitis Media with Effusion, Adenoids, *Helicobacter pylori*

## Abstract

**Background:**

Otitis media with effusion (OME) is a common disease in children. Viral or bacterial infections, allergy, adenoids, functional abnormalities of the Eustachian tube, and gastroesophageal reflux might have a possible role in the pathogenesis of OME. However, the exact pathogenesis of OME is still unsettled.

**Objectives:**

The purpose of this study was to compare *Helicobacter pylori *prevalence rates in the nasopharynx of pediatric patients with and without OME.

**Patients and Methods:**

Eighty-four patients (50 males and 34 females) who were subjected to adenoidectomy and myringotomy were included in the study group. Ninety-one patients (48 males and 43 females) who had only adenoidectomy were selected as the control group. Detection of *H. pylori* was done by polymerase chain reaction (PCR).

**Results:**

Adenoid samples were positive for *H. pylori* in 21 (25%) patients in study group and 18 (19.8%) patients in control group. In the study group, 36 (42.8%) effusion samples (otitis media) of the patients were positive for *H. pylori*. In an analysis that compared *H. pylori*–negative and –positive children, the odds ratio (OR) for the occurrence of *H. pylori* was 1.35 (95% CI, 0.66 - 2.71). The association of age with *H. pylori *positivity decreased for 1-5 years age group, (1.09; 95% CI, 0.39 – 3.05) but increased for the 6-10 years group (OR, 1.48; 95% CI, 0.61–3.58). Furthermore, the association of sex with *H. pylori* positivity decreased for the male group (OR, 1.21; 95% CI, 0.50 – 2.91), but increased in the female group (OR, 1.44; 95% CI, 0.51–0.4.05).

**Conclusions:**

Heavy colonization of *H. pylori* in adenoid tissue and middle ear might have a role in pathogenesis of this infection. For OME cases resistant to medical treatment, it might be meaningful to evaluate the patient for *H. pylori*.

## 1. Background

Otitis media is the most prevalent infection in children and the most common indication of antibacterial therapy and surgery in this group of patients (1). Besides, otitis media is the main cause of hearing loss. The cost of treatment of otitis media is a considerable figure. In the USA the expenditure of antibiotic therapy on children younger than five years of age is estimated to be $5 billion per year (2). This figure for surgery in this age group is about 600000 USD per year (3, 4). Non-purulent otitis media accompanied with middle ear effusion is called otitis media with effusion (OME) or serous otitis media.

Chronic otitis media is characterized with middle ear effusion for at least three months without clinical manifestation. OME is one of the most common ear diseases during childhood (5). It is believed that gastroesophageal reflux disease (GERD) is a predisposing factor in most upper respiratory route problems including pharyngoesophagitis, croup, oropharyngeal dysphagia, rhinosinusitis, otalgia, and some types of otitis media (6). The relationship between chronic tubotympanic disorders and GERD has been determined but the underlying mechanism is still unknown (6).

GERD is a common physiologic event in newborns and infants and its rate declines by the end of the first year of life. Refluxed materials of the stomach can reach the middle ear probably due to pre mature angle of Eustachian valve, which is a characteristic of infant skull anatomy. GERD may lead to inflammation of nasopharynx, Eustachian valve, imbalance in pressure of Eustachian tube, and malfunction of the valve (7).

*Helicobacter pylori* is a microaerophilic, Gram negative, spiral microorganism that was discovered by Marshal and colleagues in 1983 from ulcers of the stomach (8). In the short term, *H. pylori* is the etiology of antral gastritis and stomach ulcer while in the long term, infection with *H. pylori* may lead to carcinoma at the site of ulcer and mucosa-associated lymphoid tissue (9-13). In children younger than five years of age, manifestations of gastritis and stomach ulcer are not clear and only recurrent gastric pain is recordable (9-13). Polymerase chain reaction-based methods have demonstrated sequence specific DNA of *H. pylori* in nearly 80% of cases (14). 

Recently, *H. pylori* has been detected in patients with hypertrophy or chronic infection of adenoids or tonsillectomy suggesting that tonsils and adenoids act as reservoirs of the bacterium (15, 16). Moreover, *H. pylori* have been detected in the mucosa of sinuses of patients with chronic sinusitis suggesting the possible role of *H. pylori* in pathogenesis of chronic sinusitis (17, 18). Considering the close relationship between GERD and tubotympanic disorders, the probable role of *H. pylori* in OME was investigated in this study.

## 2. Objectives

In this study, *H. pylori* infection of adenoid tissue and serous aspiration obtained from infants with OME were investigated and the results were compared to samples collected from patients with hypertrophy of adenoid without OME.

## 3. Patients and Methods

### 3.1. Patients and setting

Patients were allocated in two groups: the study group included children with hypertrophy of adenoid and chronic OME and the control group included infants with hypertrophy of adenoid without OME. The study protocol was approved by Ahvaz Jundishapur University of Medical Sciences Ethical Committee and all participants signed the informed consent prior to the study.

Inclusion criteria were children with one to six years of age operated for their adenoid hypertrophy with an adenoid size of 3 cm, the presence of middle ear fluid in at least one ear on physical exam for at least three months, and OME causing an average air-bone gap of greater than 20 db. Children with other health problems such as cleft palate, neurological delay, cerebral palsy, Down syndrome, velocardiofacial syndrome, and primary ciliary dyskinesia were excluded from the study. 

### 3.2. Methods

The diagnosis of adenoid hypertrophy was based on observing the clinical manifestation and lateral view radiographic images of the nasopharynx soft tissue. Detection of chronic OME was based on observation of its clinical signs or tympanogram type C2 or B up to three months after treatment. All patients were subjected to adenoidectomy. Besides, myringotomy was performed in the study group and in case of a presence of the effusion in the middle ear, tympanoplasty tube (VT) was placed for the patient. Samples collected from OME and adenoidectomy were stored at -20˚C until extraction of DNA. *H. pylori* DNA was extracted by Roche DNA Template Purification Kit (Germany). Extracted DNA was used as a template in PCR for amplification of a 650 bp amplicon. PCR Primers 93089 and 93261 were selected from consensus regions of the two available CAgA gene sequences 400 bp product according to a previous published (18). 

The mixture was denatured initially for five minutes at 94°C, followed by 30 cycles of amplification in a PCR processor. Each cycle included a denaturing step at 94 °C for one minute, an annealing step at 45 °C for one minute and a chain elongation step at 72 °C for 90 seconds. The PCR product was loaded onto 1.5% agarose gel and electrophoresed for about one hour at 110 volts. The gel was stained with ethidium bromide and the amplicon was visualized under UV transilluminator (Vibrant, France). The size of the amplicon was compared with a DNA molecular marker (Roche, Germany). The nature of the study, method of operation, and preparation steps before and after operations were discussed with the patients or their parents and an informed consent form was obtained for use of their OME and adenoid tissue for PCR experiment.

### 3.3. Statistical Analysis

The software (SPSS version 17) was used to analyze the data. In addition to descriptive analyses, a chai-square test was performed for testing the *H. pylori* status. We used unconditional logistic regression analysis to estimate crude and adjusted odds ratios (ORs) with 95% confidence intervals (CIs) for reported occurrence of *H. pylori*, defined by yes or no. The following covariates were adjusted for sex and age.

## 4. Results

The study group including 84 patients (50 males, 34 females) was subjected to adenoidectomy and myringotomy. The control group included 48 males and 43 females. For the control group, only adenoidectomy was performed and PCR of 18 (19.8%) samples was positive for *H. pylori*, 11 (22.9%) samples were from males and 7 (16.3%) samples females. Eight patients (18.6%) were in age group of 1-5 years and 10 (20.8%) patients belonged to the age group of 6-10 years. In the study group, 21 (25%) samples were positive for *H. pylori* (12 males and 9 females). Eight patients were between one to five years of age and 13 patients were in the age group of 6-10 years.

PCR of middle ear samples of 36 (42.8%) patients in the study group were positive for *H. pylori* (25 male and 11 female). Fifteen (41.7%) patients were in the age group of 1-5 and 21 patients (43.8%) in the age group 6-10 years (Table 1). Odds ratio of infection of OME with *H. pylori* in patients with hypertrophy of adenoid accompanied by OME was 6.88 (P<0.01). This ratio for males was 7.66 (P < 0.05) and for females this was 8 (P < 0.01). Odds ratio was calculated to be 6.33 (P < 0.03) for the age group of 1-5 and 7.27 (P < 0.05) for the age group of 6-10 years.

**Figure 1.**
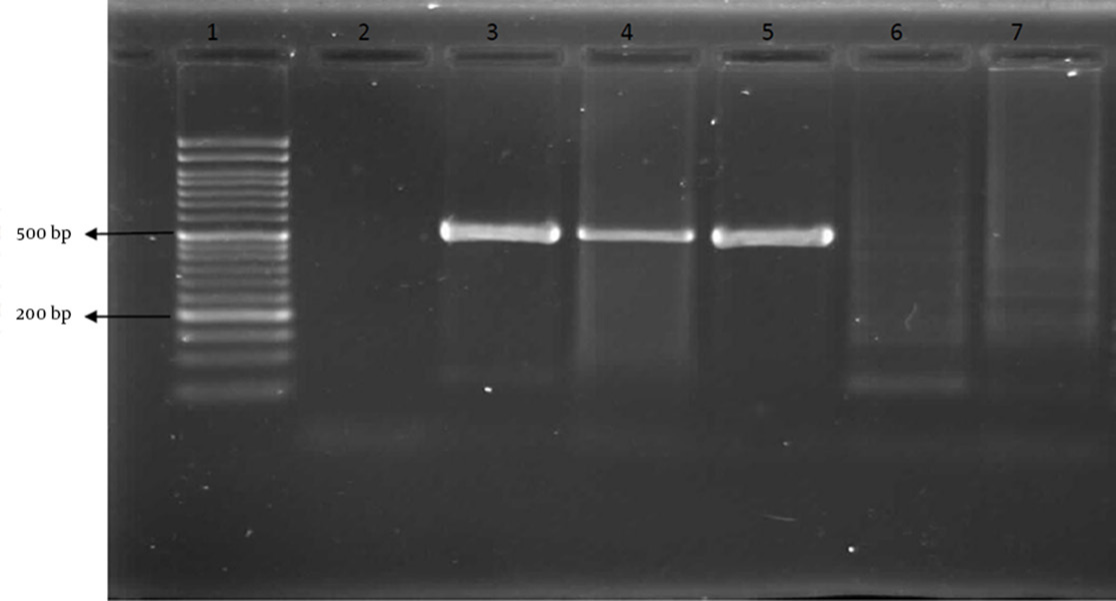
PCR Positivity for *H. Pylori* Lanes are numbered in the following order from 1 to 7: ladder (50 bp), negative control, positive control, case HP-positive (520 bp), control HP-positive (520 bp), case HP-negative, and control HP-negative.

Finally, the odds ratio for infected OME with *H. pylori* in patients with hypertrophy of adenoid was calculated to be 1.35 (P < 0.05). PCR results of the study and control groups are shown in Figure 1. In an analysis that compared children with negative and positive results for the *H. pylori *test, the OR for the occurrence of *H. pylori* was 1.35 (95% CI, 0.66 - 2.71; Table 1). The association of age with *H. pylori* positivity decreased for the age group of 1-5 years (OR, 1.09; 95% CI, 0.39 – 3.05; Table 1) but increased in the 6-10 years of age group (OR, 1.48; 95% CI, 0.61–3.58; Table 1). Furthermore, the association of sex with *H. pylori* positivity decreased for the male group (OR, 1.21; 95% CI, 0.50 – 2.91; Table 1), but increased for the female group (OR, 1.44; 95% CI, 0.51–0.4.05; Table 1). 

**Table 1.** Relationship Between *H. pylori *and Age and Sex, by Multivariate Analysis Among Study Groups

Variables	Number of Cases		OR ^a^	95% CI ^a^	P Value
	Case (n = 84)	Control (n = 91)			
***H. Pylori***					
Positive	21	18	1.35	0.66 - 2.71	0.258
Negative	63	73	Ref.	-	-
**Age**					
1-5 years	8	8	1.09	0.39 – 3.05	0.536
6-10 years	13	10	1.48	0.61 – 3.58	0.256
**Sex**					
Male	12	11	1.21	0.50 – 2.91	0.417
Female	9	7	1.44	0.51 – 4.05	0.333

^a^ Abbreviations: CI, confidence interval; OR, Odds ratio

## 5. Discussion

In this study, *H. pylori* infection of adenoid tissue and serous aspiration obtained from children with chronic OME was investigated and the results did not find a strong correlation between *H. pylori* infection and OME. *H. pylori *is a Gram negative bacterium and the etiologic agent of some gastrointestinal and extra gastrointestinal diseases (19). Colonization of *H. pylori* has been found in dental plaques, saliva, tonsils, sinus mucosa, and adenoids. Recently, the role of GERD and *H. pylori *in the development of OME has been emphasized (20, 21).

Amicroaerophilic environment is necessary for optimum growth of *H. pylori*, a condition available in middle ear during OME. Mechanism of colonization of *H. pylori* in the mucous of the stomach has not been determined. Acidic pH of the stomach is not in favor of growth of this bacterium. For survival, *H. pylori* penetrate inside the stomach and the epithelium mucous layer. This layer is resistant to penetration of acid of the stomach and plays as a supportive layer for the bacterium. The pH of the stomach is 1-2 while it is 7.4 inside the mucous layer and 7.9 in the middle ear during OME (22-24).

Mucous metaplasia and goblet cells hyperplasia might occur after *H. pylori* infections. Similar outcome is seen after OME (24). In both inflammatory reactions, lymphocyte infiltration exists. *H. pylori* can be found in the middle ear. In our study, this bacterium was detected in the middle ear of 42.6% of patients with OME. In a research conducted by Yilmaz T et al. and Yilmaz MD et al. 18 patients with adenoid hypertrophy and OME were assessed and *H. pylori* was found in 12 (67%) patients and 16 (47%) ears out of 34 ears were infected by this bacterium, respectively (25, 26). Agidir et al. tested 30 patients with adenoid hypertrophy and OME and urease test results were positive in 66.6% of OME samples (27). This rate ranged from 16.3 to 35.5 when PCR was performed (28). In our study, 25% of patients in the study group and 19.8% of patients in the control group had positive results for *H. pylori*. Agirdir reported positive results in 33% and 26% of study and control groups, respectively. The results of other studies ranged from 0 to 64%, depending on the method of detection of *H. pylori (*27*)*.

Our results showed that *H. pylori* infection of the middle ear was twice more common than infection of adenoid tissue. Perhaps the condition of the middle ear is more favorable for growth of this bacterium. Inoculation of *H. pylori* may find its way towards the middle ear either hematogenously or via refluxed materials in GERD. GERD is a usual problem, especially in adenoid hypertrophy. Carr et al. showed that out of 95 infants younger than two years of age who had undergone adenoidectomy, 42% had GERD (29). Farhadi et al. found *H. pylori* in adenoid tissues of 15% children with adenoid surgery (18).

Stomach secretions containing *H. pylori* enter the oral cavity and subsequently the bacterium colonizes in dental plaques, tonsils, and adenoid tissues. From these regions, the bacterium ascends to the middle ear and paranasal sinuses directly or via GER and causes pathological changes with an unknown mechanism. Currently, there is no standard protocol for treatment of OME. Most of the OME patients do not respond to therapy and have to undergo surgery. Surgery may alter the microaerophilic environment of the middle ear and inhibit colonization of *H. pylori* in this organ.

### 5.1. Conclusions

In this study, the existence of *H. pylori* in the middle ear was investigated. We could not find a strong correlation between *H. pylori* infection and OME; hence, the pathogenesis of this bacterium in OME awaits more investigations.
